# Characterization of the Roles of Vimentin in Regulating the Proliferation and Migration of HSCs during Hepatic Fibrogenesis

**DOI:** 10.3390/cells8101184

**Published:** 2019-10-01

**Authors:** Pei-Wen Wang, Tung-Ho Wu, Tung-Yi Lin, Mu-Hong Chen, Chau-Ting Yeh, Tai-Long Pan

**Affiliations:** 1 Department of Medical Research, China Medical University Hospital, China Medical University, Taichung 40447, Taiwan; pwwang5105@hotmail.com; 2 Division of Cardiovascular Surgery, Veterans General Hospital, Kaohsiung 81362, Taiwan; thwu@vghks.gov.tw; 3 Department of Chinese Medicine, Chang Gung Memorial Hospital, Keelung 20401, Taiwan; tungyi30@cgmh.org.tw; 4 School of Traditional Chinese Medicine, Chang Gung University, Taoyuan 33302, Taiwan; 5 Department of Psychiatry, Taipei Veterans General Hospital, Taipei 11217, Taiwan; kremer7119@gmail.com; 6 Department of Psychiatry, College of Medicine, National Yang-Ming University, Taipei 11221, Taiwan; 7 Liver Research Center, Chang Gung Memorial Hospital, Taoyuan 33375, Taiwan; chautingy@gmail.com; 8 Chinese Herbal Medicine Research Team, Healthy Aging Research Center, Chang Gung University, Taoyuan 33302, Taiwan; 9 Research Center for Chinese Herbal Medicine and Research Center for Food and Cosmetic Safety, College of Human Ecology, Chang Gung University of Science and Technology, Taoyuan 33303, Taiwan

**Keywords:** hepatic stellate cells, hepatic fibrosis, vimentin, Rho, ERK, AKT

## Abstract

The activation of hepatic stellate cells (HSCs) manifested as proliferation and migration is the pivotal event involved in liver fibrogenesis. The vimentin network, an intermediate filament (IF) system, is one of the critical cascades by which the cell morphology, growth, and motility are modulated. However, the vimentin-mediated cytoskeletal cross talk, as well as the signaling transduction, which further coordinates the cellular responses during hepatic fibrogenesis, is poorly understood. In the current study, both messenger RNA (mRNA) and the vimentin protein were significantly increased in a time-dependent manner in the dimethylnitrosamine (DMN)-exposed liver. In particular, vimentin was highly expressed in the activated HSCs. Again, the overexpressed vimentin was observed in the plasma samples derived from patients with hepatic fibrosis/cirrhosis, suggesting that vimentin may be a key factor in regulating the progression of liver fibrosis. Meanwhile, vimentin knockdown suppressed the migratory propensity, provoked morphological changes, and disturbed the focal adhesions in the HSCs due to the breakdown of associated cytoskeletal proteins. Western blotting showed that vimentin deletion inhibited proliferating cell nuclear antigen (PCNA) and arrested the Rho GTPase family, thereby impairing the HSCs’ growth as well as motility. The phosphorylated extracellular-signal regulated kinase (ERK) and AKT signals were also notably reduced in response to the silence of vimentin. Inhibitors of selected signaling pathways suppressed the migration and differentiation of activated HSCs by regulating specific serine phosphorylated sites on vimentin. Taken together, these findings revealed a novel mechanism of vimentin through which various signaling pathways controlled the proliferation, differentiation, and movement of the HSCs via the ERK/AKT and Rho cascades.

## 1. Introduction

Hepatic fibrosis, the hallmark feature related to the failure of liver function, is characterized by the excessive deposition of extracellular matrix (ECM) proteins. Ongoing fibrosis may result in irreversible cirrhosis and an increased risk of hepatoma [1,2,3,4]. Currently, liver fibrogenesis has become an important public health issue worldwide due to the high prevalence of chronic liver diseases [5,6,7]. In this regard, anti-fibrotic administration should be urgently developed to control or even inhibit disease progression.

Hepatic stellate cells (HSCs), located in the space between the hepatocytes and the sinusoidal endothelium, play a critical role during the progression of liver fibrosis. Specific cytokines and molecules such as transforming growth factor-β1 (TGF-β1) and platelet-derived growth factor (PDGF) stimulate the HSCs to undertake the phenotypic switch from quiescent retinoid-storing cells to proliferative and fibrogenic cells, which are thought to play a key role in fibrosis of the liver [8,9,10]. Therefore, the suppression of cell growth and the migration of HSCs may be a promising strategy for reversing early fibrosis, although the intracellular signals regulating the changes in HSCs remain unclear.

One of the earliest events associated with HSC activation is cytoskeleton remodeling, which contributes to cell division and movement [11,12]. Coordinated movement is the result of the ability of a cell to spread protrusions and form adhesions at the leading edge, translocate the cell body, and eventually disconnect from the substrate. Of note, vimentin, a member of the intermediate filament (IF) protein family, helps to stabilize focal adhesion, which governs cell migration. It also performs as a signal transducer from the ECM to the nuclei [13,14]. Moreover, vimentin has a great number of phosphorylation sites that influence the assembly status and the architecture of the cellular filaments. The vimentin network recruits rapid phosphorylation and dephosphorylation that regulate integrin-mediated cell adhesion and facilitate directional cell motility [15,16]. In keeping with such roles, identification of the coordinated proteins and molecules modulated by vimentin might reveal the IF-mediated cytoskeleton cross talk associated with liver fibrosis.

Significant evidence has shown that HSC migration is essential for liver fibrogenesis and that preventing the cell migration could notably suppress the disease progression [17]. Activation-related morphological changes and migration in the HSCs are induced by liver injury, although the associated signaling mechanisms are still unclear. The Rho GTPase family of proteins, including Cdc42, RhoA/B/C, and Rac1, trigger and control specific signal transduction pathways that initiate and regulate cytoskeletal dynamics, cell proliferation, and cell movement via the activation of the PAK serine/threonine kinases [18,19]. Herein, we investigated how Rho proteins and their signaling pathways or the vimentin-mediated regulation of downstream cascades are involved in the pathological remodeling of HSCs.

Through our research to better understand the regulatory mechanisms of HSC migration in liver fibrogenesis, we verified the hypothesis that vimentin may influence cytoskeletal reorganization and cell migration in HSCs through Rho GTPase signaling, particularly RhoA.

## 2. Material and Methods

### 2.1. Materials

Specific antibodies to GAPDH, vimentin, PCNA, collagen I, filamin, vinculin, and talin were purchased from Santa Cruz Biotech (Dallas, TX, USA). Polyclonal antibodies to PPARγ, α-SM-actin, and β-actin were obtained from abcam (Cambridge, UK). Polyclonal antibodies to Rho A, Rho B, Rho C, Rac1/2/3, Cdc42, phospho-Rac1/cdc42, AKT, phospho-AKT, ERK, phospho-ERK, phospho-Vimentin^Ser39^, phospho-Vimentin^Ser56^ and phospho-Vimentin^Ser83^ were purchased from Cell Signaling (Danvers, MA, USA). U0126, Y27632, LY294002 was obtained from Cayman Chemical (Ann Arbor, Michigan, USA). DMN was purchased from Sigma-Aldrich (St. Louis, MO, USA).

### 2.2. Animals

Male Wistar rats weighing 200–225 g were purchased from Lasco Co. (Taipei, Taiwan) and were randomly divided into two groups of six each [control (saline treated) and DMN treated]. In the DMN-induced fibrosis group, rats were injected intraperitoneally with DMN (10 mg/kg body weight; Sigma) for three consecutive days per week for four weeks [20,21]. The control group was applied with only saline. At the end of the fourth week, all of the rats were sacrificed. The liver tissues were excised and the specimens were immediately fixed in 10% neutral buffered formaldehyde for pathological and immunohistochemical studies. The Committee on Research Involving Animal Subjects of the Chang Gung University, Taiwan, has approved the study.

### 2.3. Analysis of Transcripts of α-SMA, Vimentin, and Procollagen Genes

Total RNA was isolated from liver tissue, and single-stranded cDNA synthesis was performed on 10 μg of total RNA by a complementary DNA synthesis system for the RT-PCR according to the manufacturer’s instructions (Invitrogen, Waltham, MA, USA). The primers utilized for the PCR experiments are listed in the following. α-SMA: 5′-TTCGTTACTACTGCTGAGCGTGAGA-3′, 5′-AAAGATGGCTGGAAGAGGGTC-3′; vimentin: 5′-ATGGCTGCCAAAGTGTT-3′, 5′-CTGGGGGAGCTGGAGG-3′; Col I: 5′-TACTACCGGGCCGATGATGC-3′, 5′-TCCTTG GGGTTCGGGCTGATGTA-3′; Col III: 5′-CCCCTGGTCCCTGCTGTGG-3′, 5′-GAGGCCC GGCTGGAAAGAA-3′; β-actin: 5′-TGTTACCAACTGGGACGACA-3′, 5′-CTCTCAGCT GTGGTGGTGAA-3′ [21,22]. The condition consisted of denaturing at 94 °C for 1 min, annealing at 53 °C for 1.5 min, and extending at 72 °C for 2.5 min. Each PCR product was resolved on a 1.5% agarose gel incorporated with ethidium bromide. Transcript intensities were revealed as digitalized images using a high-resolution scanner (Syngene, Cambridge, UK). The β-actin transcript was used as a loading control to normalize the concentration of cDNA in each sample. Take a ratio of a net band value over the net loading control of that lane after background subtraction for quantification.

### 2.4. Western Blot Analysis

Equal amount of lysated protein was separated on 6%, 12% or 15% denatured gels, respectively and transferred to membranes. Next, the blots were incubated with specific primary antibody overnight at 4 °C after blocking and further incubated with a peroxidase-labeled anti-mice or -rabbit IgG for 2 h. After washing with TBST several times, enhanced chemiluminescence (PerkinElmer, Waltham, MA, USA) was used for signal detection. The band intensity was quantified using GeneTools Image Software (version 4.03, Syngene). GAPDH and β-actin were used as the loading controls [20]. Take a ratio of a net band value over the net loading control of that lane after background subtraction for quantification.

### 2.5. Histology and Immunohistochemistry

The liver tissue fixed by 5% neutral buffered formalin was immersed in paraffin and then sliced into 5 μm sections. The sample slices were stained with hematoxylin–eosin (H/E) and Masson’s trichrome (MT) for a histological assessment. Immunohistochemistry with vimentin was applied to specimens as previously described [20,23]. The histological changes were observed by using optical microscopy (Olympus BX51, Tokyo, Japan) in non-consecutive, randomly chosen 400× histological fields. The digital photomicrographs were then processed with DP-72.

### 2.6. Clinical Cases

To reveal the clinicopathological significance and relevance of vimentin expression in hepatic fibrosis, we have applied a separate cohort of 55 subjects (22 controls and 33 patients with liver fibrosis) under approval of Institutional Review Board, Chang Gung Memorial Hospital, Taiwan, plasma samples were retrospectively retrieved from the serum bank, Liver Research Center, Chang Gung Memorial Hospital for a study attempting to correlated biochemistry, tissue histology, and harmonic microscopy characteristics for liver fibrosis in hepatitis B patients. All patients included had previously received liver biopsy for evaluation of hepatitis activities and liver fibrosis which is classified by “ISHAK” score [24]. The protein amount was determined and normalized by using the Bradford Protein Assay Kit (AMRESCO, Solon, OH, USA)). Western blotting assays were conducted and quantified with GeneTools Image Software (version 4.03, Syngene). All experiments were technically repeated three times.

### 2.7. Cell Culture

The immortalized rat myofibroblast cell line HSC-T6 was a kind gift of Dr. Scott L. Friedman (Mount Sinai School of Medicine, New York, NY, USA). The HSC-T6 cells were maintained in DMEM medium containing 10% FBS at 37 °C in a humidified atmosphere of 5% CO2.

### 2.8. Gene Silencing by Small Interfering RNA

HSC-T6 cells were plated onto six-well plates (1 × 10^5^ cells/well), maintained in antibiotic-free medium for 24 h, and transfected with a mixture containing Opti-MEM, 8 μL/well Lipofectamine 2000 (Invitrogen), and either 0.5 μg/well scrambled siRNA (mock) or a vimentin siRNAs (smart pool; Invitrogen) for 6 h [25,26]. The sequences of these siRNAs are available from the manufacturer. After another 48 h culture with DMEM medium containing 10% FBS under transfection, cells were evaluated by the Wound-migration assay, Immunofluorescence and Western blot. Determination of cell proliferation and apoptosis by the flow cytometry and western blot was described previously [26].

### 2.9. Wound-Migration Assay

A wound-migration assay was performed as described by Van Lonkhuyzen et al. with slight modifications [27]. HSC-T6 cells transfected with or without siVIM were grown in 35-mm culture dishes to 95% confluence. A wound was formed using a 200 μL pipette tip to clear the cell monolayer, and the boundary of the wound was marked. Cells were then washed three times with PBS and incubated for 24 h at 37 °C under a 5% CO2 atmosphere. After incubation, cell migration was measured by counting the number of cells that migrated into the clear space using an Olympus microscope (IX71) at 20× fitted with an ocular grid. Results presented are the mean of four random fields of wounds sampled from three independent experiments. The areas of cell migration were determined by dividing the mean number of cells that moved from the edge to the wounded area by cells that moved from the edge in the control culture.

### 2.10. Immunofluorescence

HSC-T6 cells (mock or siVIM) were fixed in ice-cold methanol for 10 min at 4 °C. After washing in PBS, cells were permeabilized with 0.1% Triton-X100 in PBS for 10 min. After blocking with 1% BSA in PBS, cells were then incubated with primary antibodies and rinsed three times in PBS. The cells were subsequently exposed to a rhodamine conjugated secondary antibody. After incubation, cells were rinsed in PBS three times and nuclei were counterstained with DAPI for 1 min. After washing three times, the cells were maintained with mounting medium and observed by Olympus IX71 fluorescence microscope with DP72 PhotoImage system [25].

### 2.11. Statistical Analysis

The statistical analysis of the mean values was carried out with the ANOVA followed by Bonferroni post-hoc analysis to reveal what pairs of group means show differences with Prism software (v5.0, Prism GraphPad, San Diego, CA, USA). Bar charts are presented as the mean ± SD and *p* values from the post-hoc tests are included in the text and figure legends as conducting paired comparisons.

## 3. Results

### 3.1. Liver Pathological Changes and Vimentin Expression Induced by DMN Administration

**Figure 1:**
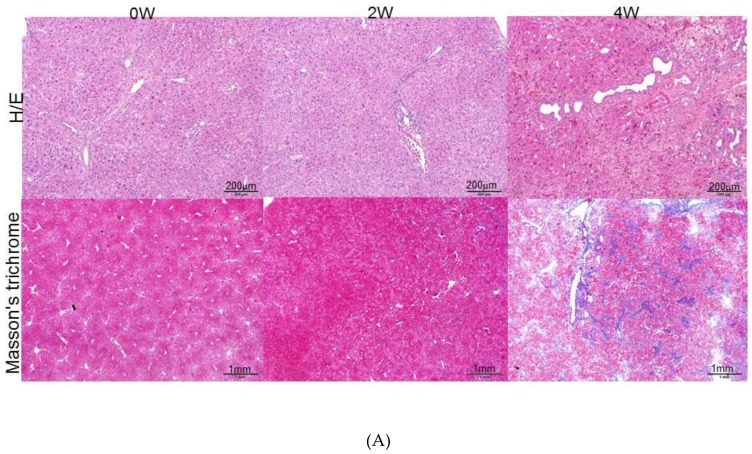

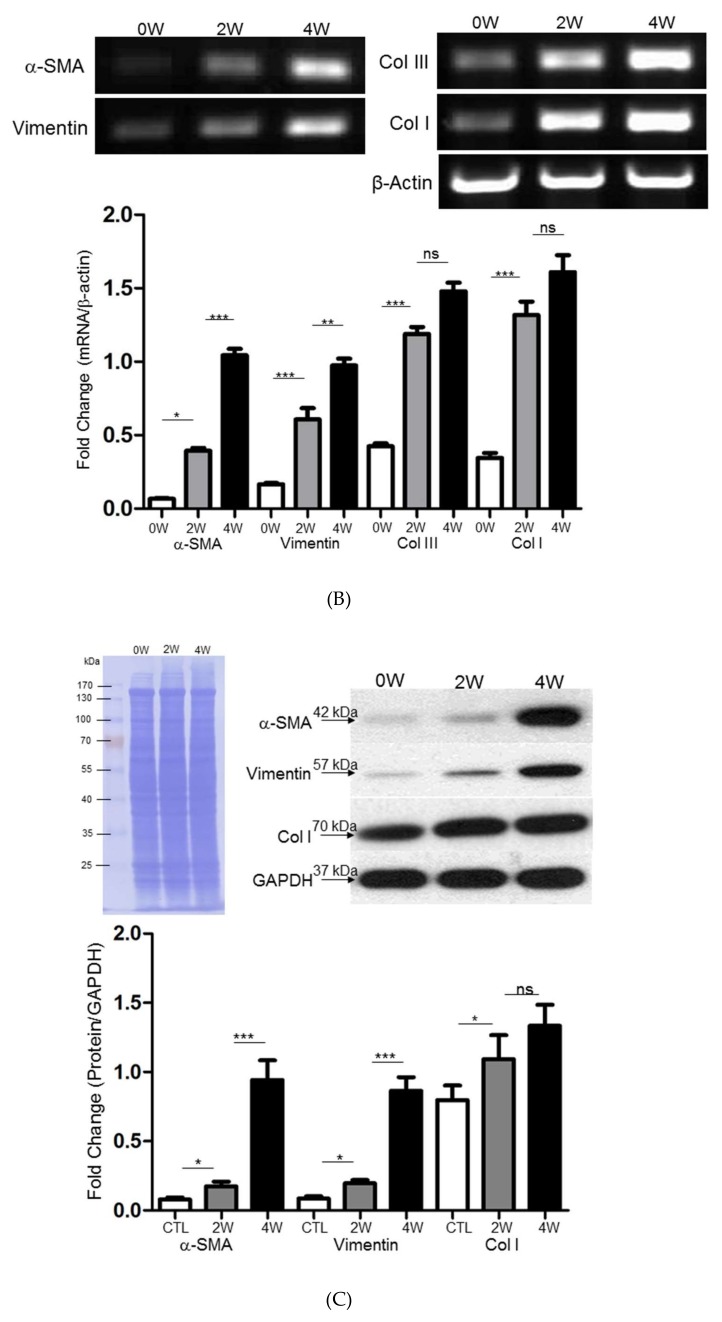

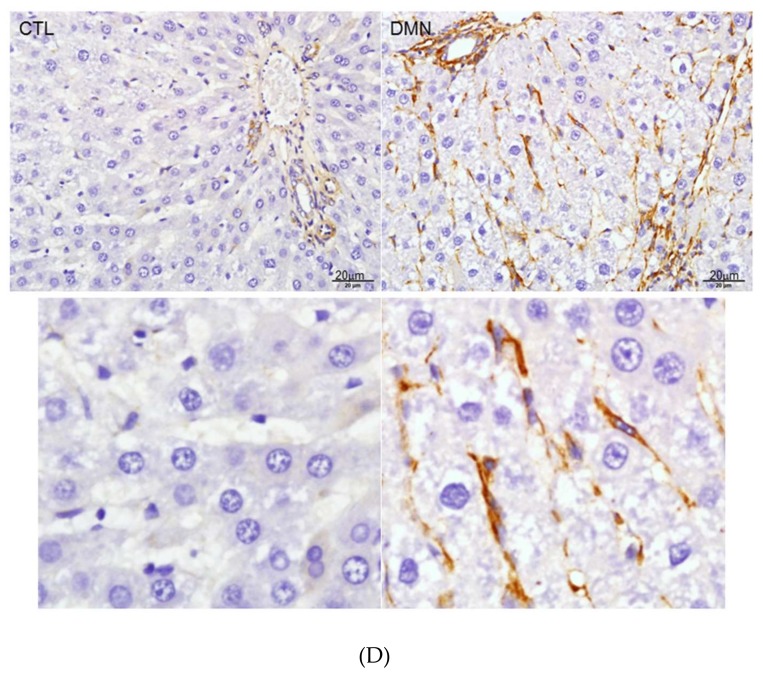
(**A**) Histologic examination of rat liver at zero week (0W), two weeks (2W), and four weeks (4W). Upper panels: Hematoxylin–eosin (H/E) staining indicated necrosis of hepatocytes and infiltrated lymphocytes. Lower panels: Masson’s trichrome staining of rat liver tissues. The images indicated accumulation of collagen around portal tracts as blue images. (**B**) Validation of α-smooth muscle actin (α-SMA), vimentin, collagen type III (Col III), and collagen type I (Col I) expression by RT-PCR after treatment of dimethylnitrosamine (DMN). β-Actin was used as an internal control. The quantified results were indicated by the bar chart and represent the mean ± SD of three independent experiments (* *p* < 0.05, ** *p* < 0.01, *** *p* < 0.001, ns: no significance). (**C**) Confirmation of change in extracellular matrix (ECM) protein level after DMN application. Protein expression of α-SMA, vimentin, and Col I was assessed by a Western blot analysis. Glyceraldehyde-3-phosphate dehydrogenase (GAPDH) was used as an internal control. Polyvinylidene difluoride (PVDF) membrane stained with Coomassie blue R-250 was utilized to perform loading amount of proteins. The quantified results were presented by the bar chart (* *p* < 0.05, *** *p* < 0.001, ns: no significance.). The results are representative of the rats used in each group (*n* = 6). (**D**) Immunohistochemical study of vimentin expression in representative liver tissues obtained from samples treated with or without DMN at four weeks (4W). The regions with differently expressed vimentin were shown by brown color. The lower panels presented the zoom figures.

As shown in the upper panels of Figure 1A, with the use of hematoxylin–eosin (H/E) staining, the control sample showed intact lobular architecture, whereas the application of dimethylnitrosamine (DMN) for two weeks caused necrosis of the hepatocytes, inflammatory infiltration, and early liver fibrogenesis. Four-week exposure to DMN resulted in severe hepatic injury, which manifested as marked fibrosis with a huge amount of accumulated collagen. Masson’s trichrome stain demonstrated that DMN at four weeks induced severe liver fibrosis where a large amount of collagen was accumulated (Figure 1A, lower panels) with respect to the control sample. Semi-quantification of RNA expression analysis also indicated that the mRNA expression of α-smooth muscle actin (α-SMA), collagen proteins such as collagen type I (COL I) and collagen type III (COL III), and vimentin was increased in a time-dependent manner after DMN treatment (Figure 1B). Consistent with the transcription results, the protein levels were gradually enhanced from week zero to week four following DMN administration (Figure 1C), which explains the development of hepatic fibrogenesis. Previous reports have shown that DMN would stimulate quiescent HSCs into proliferating myofibroblast-like cells, subsequently leading to liver fibrogenesis [20,28]. In the current study, histological changes in the liver tissue of rats were evaluated. Well-developed hepatocytes arranged in an orderly manner were identified in the normal control, and HSCs exhibited a dendrite-like shape encircling the sinusoids. Conversely, DMN applied samples showed serious hepatic injury characterized as activation of HSCs with extensive cytoplasmic fibers, massive necrosis of the hepatocytes, and inflammatory infiltration. Meanwhile, the location of vimentin was also confirmed by using immunohistochemistry, and a strong vimentin signal was predominantly detected in activated HSCs in the presence of DMN, suggesting the strong role of vimentin in directing the activation of HSCs (Figure 1D).

### 3.2. Plasma Levels of Vimentin between Control and Patients with Hepatic Fibrosis/Cirrhosis

**Figure 2:**
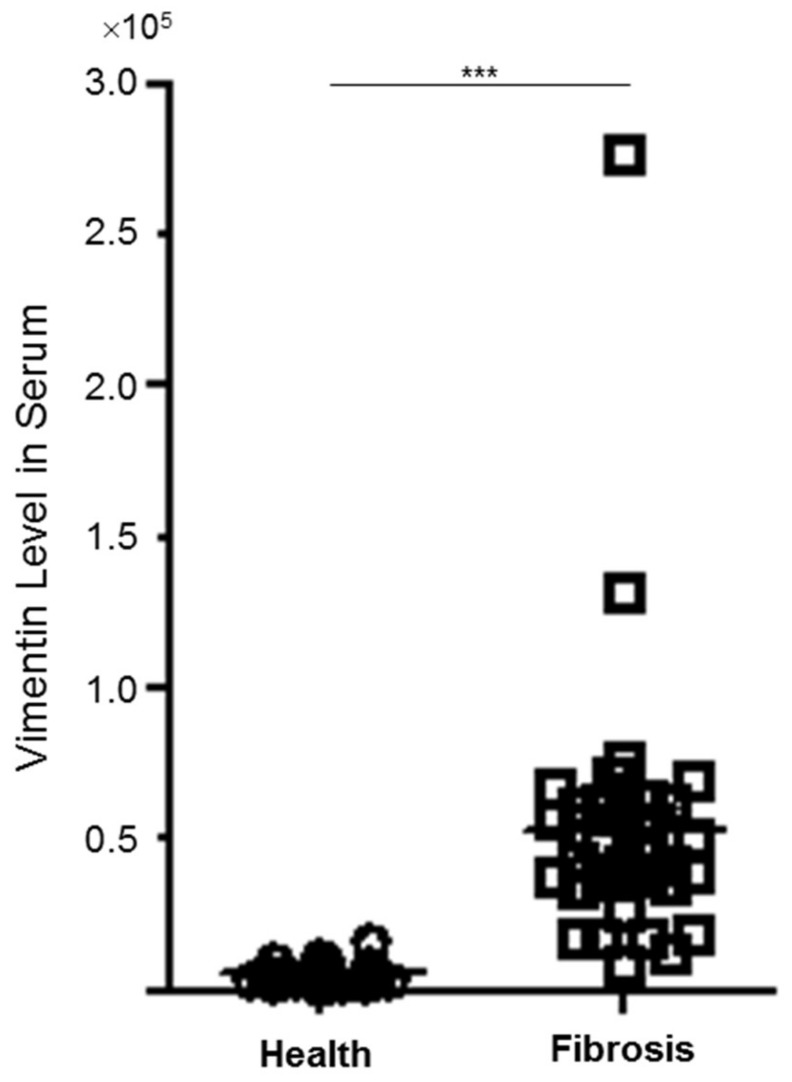
The quantitative results indicating the different levels of vimentin in clinical plasma specimens between normal control (*n* = 22) and patients with hepatic fibrosis (*n* = 33) (*** *p* < 0.001).

To further verify the role of vimentin in the progression of hepatic fibrosis, we evaluated the levels of vimentin in the clinical plasma specimens obtained from the healthy controls and from subjects with liver fibrosis/cirrhosis. Not surprisingly, the level of vimentin in the patient sample was significantly upregulated compared with that in the control group (*p* < 0.001) as shown in Figure 2, implying that vimentin may be a potential modulator in hepatic fibrosis.

### 3.3. Functional Roles of Vimentin in Regulating HSC Activation

**Figure 3:**
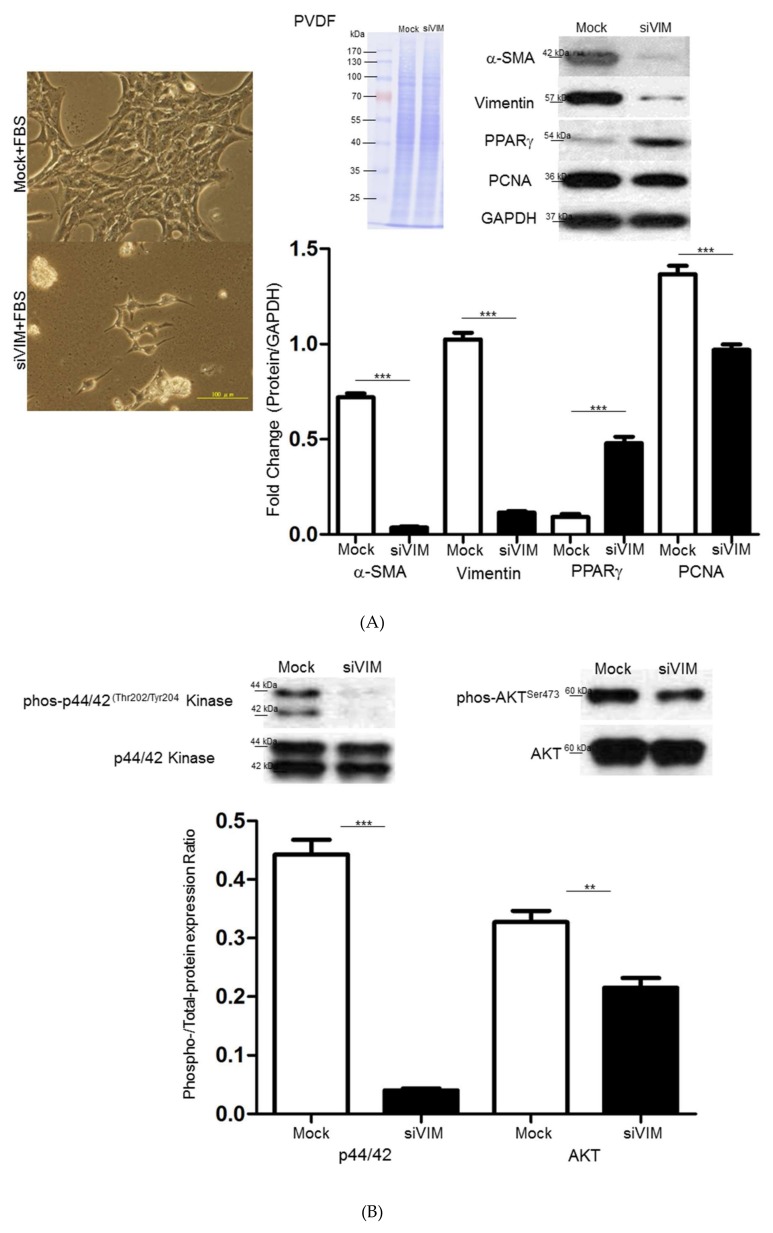

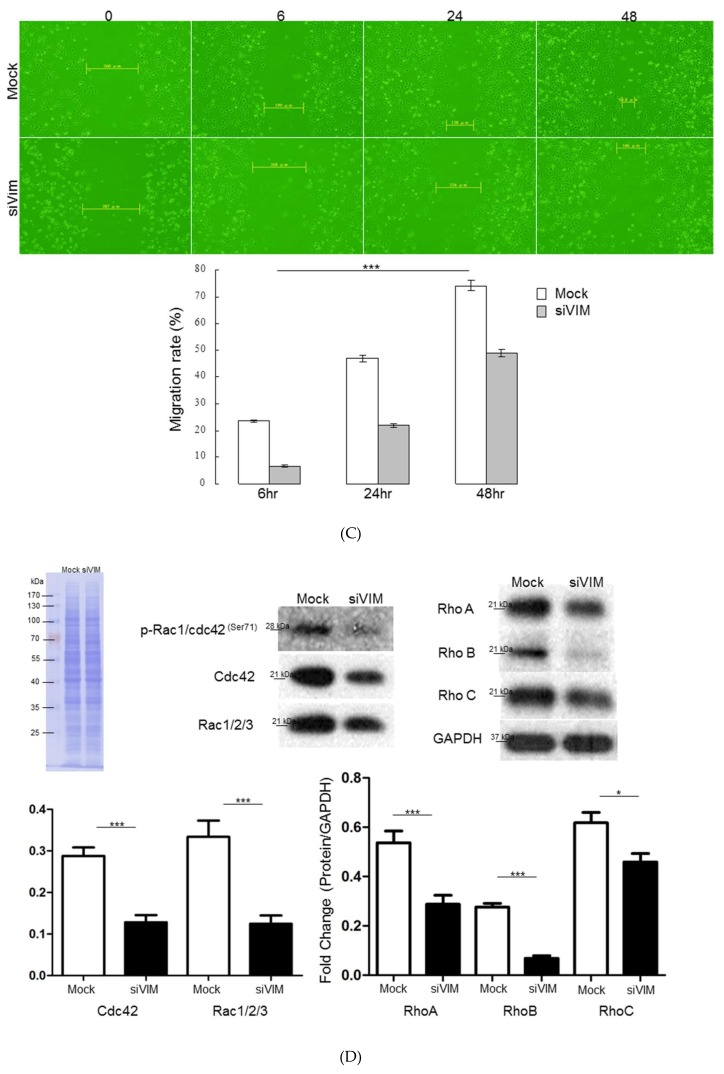
(**A**) Validation of α–SMA, vimentin, peroxisome proliferator activated receptor-γ (PPARγ) and proliferating cell nuclear antigen (PCNA) expression in hepatic stellate cell (HSC-T6 cells applied with vimentin with RNA silencing (siVIM) or without (Mock) siVIM by Western blotting. GAPDH was used as an internal control. PVDF membrane stained with Coomassie blue R-250 was utilized to perform loading amount of proteins. The quantified results were indicated by the bar chart and represented the mean ± SD of three independent experiments (*** *p* < 0.001). Morphological changes of HSC-T6 cells with or without siVIM transfection by optical microscopy were demonstrated as the left figures. (**B**) Western blot analysis for phosphorylation and total protein levels of mitogen-activated protein kinases (MAPK) and protein kinase B (AKT) in HSC-T6 cells after administrating with or without siVIM. The quantification of phosphorylation in each lane was normalized by total protein levels (** *p* < 0.01, *** *p* < 0.001). (**C**) Knockdown of vimentin retarded wound closure. Representative phase-contrast micrographs of closure of scratch-wounded confluent cultures of mock- or siVIM-transfected HSC-T6 cells at a time point immediately after wounding and 24 h post-wounding. The migration rate was calculated by the percentage (%) and indicated with the bar chart (*** *p* < 0.001). (**D**) Western blot analysis for phosphorylation and total protein levels of Cdc42, Rac1/2/3, and Rho A/B/C in HSC-T6 cells after administrating with or without siVIM. The quantification of phosphorylation in each lane was normalized by total protein levels. GAPDH was used as an internal control. The quantified results were indicated by the bar chart and represented the mean ± SD of three independent experiments (* *p* < 0.05, *** *p* < 0.001).

Peroxisome proliferator activated receptor-γ (PPARγ), a key regulator in HSC activation and phenotypic alteration [29], was tested after vimentin silence to assess the potential influence of vimentin on liver fibrosis. As demonstrated in Figure 3A, Western blot analysis revealed that the knockdown of vimentin with RNA silencing (siVIM) for 48 h after transfection successfully suppressed vimentin protein expression by more than 90% with respect to the mock control. PPARγ was significantly promoted while the proliferating cell nuclear antigen (PCNA) was obviously reduced after vimentin knockdown. These findings were consistent with the cell images in which cells transfected with siVIM showed phenotypes of quiescent HSCs and low frequency in cell division. The mitogen-activated protein kinases/extracellular signal-regulated kinase (MAPK/ERK) and AKT pathways have been confirmed to be closely related to the cell proliferation, differentiation, and migration. Here, we detected the phosphorylation of ERK1/2 and AKT by Western blotting. The data showed that siVIM treatment strongly suppressed the expression of p-ERK1/2 and p-AKT, which are considered to be the indicators of HSC activation (Figure 3B).

To further investigate the efficacy of vimentin in modulating HSC motility at the molecular level, the outcome of vimentin knockdown on HSC migration was evaluated via wound-healing assays. Scratch-wound assays on confluent monolayers demonstrated that mock-control cells migrated particularly rapidly and almost filled 80% of the space within 48 h, whereas siVIM-transfected cells repopulated the cleared space at a much slower rate and still retained a double space apart at the same time point (Figure 3C). Moreover, flowcytometry analysis indicated that application of siVIM could only elicit slight apoptosis of HSC cells (Supplementary Materials). The above-mentioned findings suggested that vimentin might modulate cell migration as an organizer that would subsequently control other cytoskeletal proteins via specific signaling pathways. Western blot analysis was performed to evaluate the changes in proteins associated with cell migration in the presence of siVIM in HSCs. Significant downregulation in levels of p-Rac1/cdc42, cdc42, Rac1/2/3, Rho A/B/C was identified in siVIM-transfected HSCs compared to the control, suggesting that vimentin is critical for cell migration due to its regulatory function associated with Rho and phosphorylated cascades (Figure 3D).

### 3.4. Verifiction of Vimentin-Dependent Regulation of Cytoskeletal Proteins

**Figure 4:**
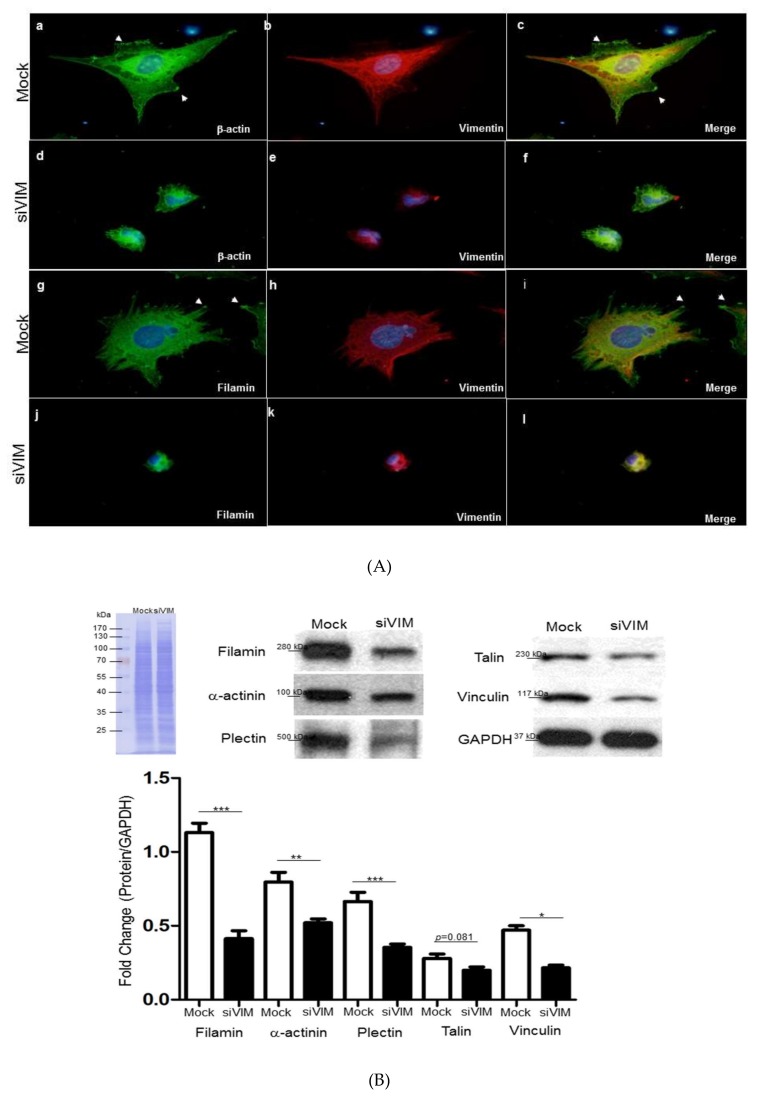
(**A**) Changes in architecture of hepatic stellate cells (HSCs) with or without siVIM transfection by immunofluorescence microscopy. Images of cells showed that vimentin knockdown caused the loss of actin stress fibers and disturbance of actin polymerization (**d,e,f**) compared to the mock samples (**a,b,c**) as indicated by arrows. The structure of local adhesion points which are constituted by filamin was destroyed in the presence of siVIM (**j,k,l**) with respect to the control (**g,h,i**). (**B**) Validation of changes in protein expression after treatment of RNA interference-mediated vimentin silencing. Protein levels of filamin, α-actinin, plectin, talin, and vinculin were assessed by a Western blot analysis. GAPDH was used as an internal control. The quantitative results were demonstrated as a bar chart (* *p* < 0.05, **p < 0.01, *** *p* < 0.001).

Next, we observed that vimentin was highly expressed at the leading edge of migrating cells and the vimentin-based scaffolding protein recruited other cytoskeletons such as actin to stabilize the structure required for HSC migration as well as related signaling transduction. Diminishment of vimentin by siVIM entirely abrogated the cytoskeletal restructuring and reorganization, leading to the cells’ deficiency in protrusion, migratory property, and various processes during HSC activation. In addition, vimentin knockdown also interfered with the complete architecture of the focal adhesion complex involved in binding cells to the extracellular matrix (ECM). As expected, siVIM destroyed the whole constitution of the focal adhesion complex, suggesting the central role of vimentin in HSCs’ architecture maintenance, contraction, and movement (Figure 4A). The aforementioned results supported the idea that vimentin should be involved in cell migration as an organizer and might also subsequently modulate other cytoskeletal proteins. Western blot analysis showed notable decreases in the levels of filamin A, α-actinin, plectin, talin, and vinculin compared to the control (Figure 4B), implying that siVIM may inhibit cell invasion by undermining the expression and reorganization of various cytoskeletal proteins. The loading control for Western blot analysis indicated that an equal amount of protein was applied.

### 3.5. Intracellular Signaling of ERK, AKT and Rho Affecting HSC Proliferation and Motility

**Figure 5:**
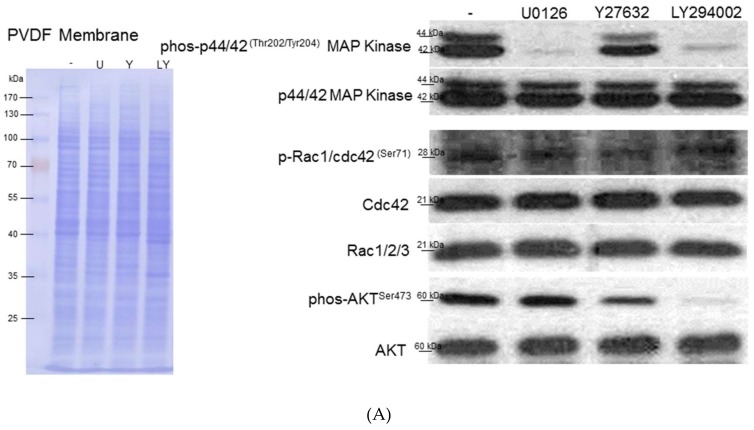

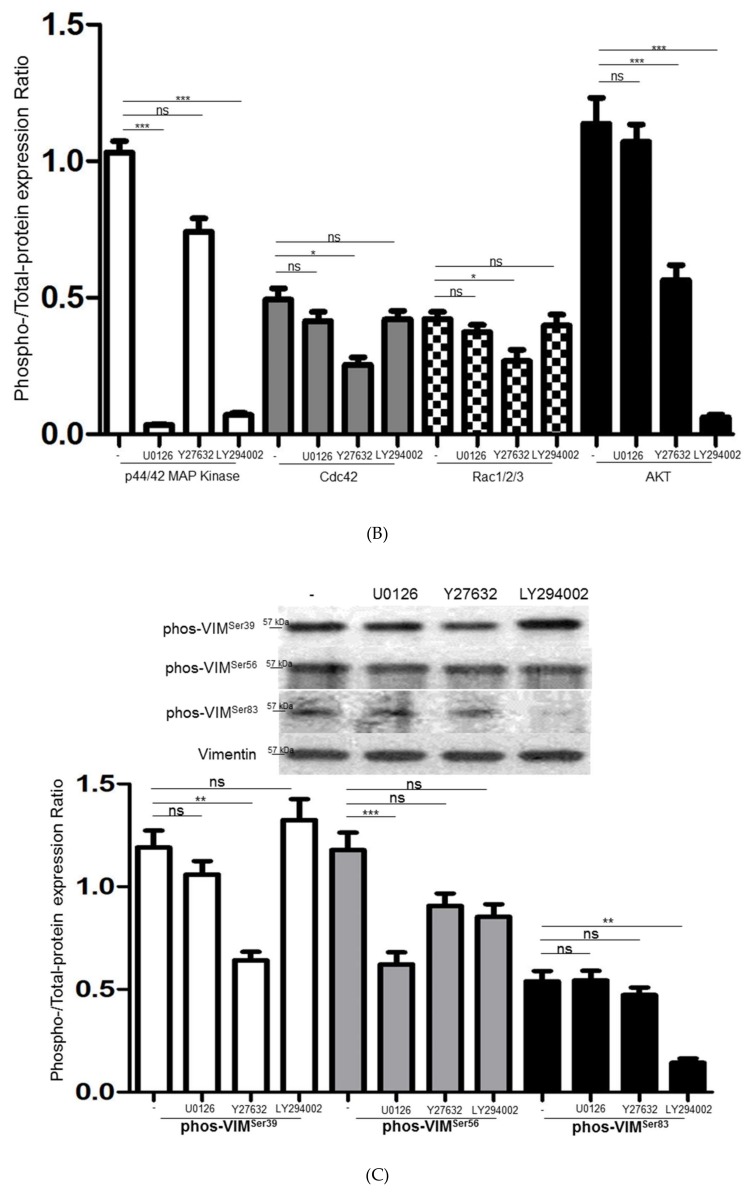

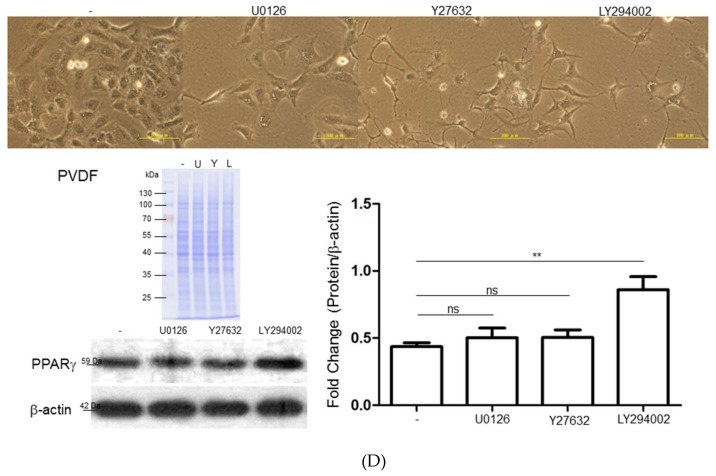
(**A**) Western blot analysis for phosphorylation and total protein levels of ERK1/2, Cdc42, Rac1/2/3, and AKT with application of different inhibitors including U0126, Y27632, and LY294002. PVDF membrane stained with Coomassie blue R-250 was utilized to perform loading amount of proteins. (**B**) The phosphorylation levels were normalized by total protein levels and the corresponding results represented the mean ± SD of three independent experiments (*p < 0.05, *** *p* < 0.001, ns = no significance). (**C**) The expressed levels of various phosphorylated sites of vimentin were modulated under treatment of specific inhibitors such as U0126, Y27632, and LY294002. The quantified results were indicated by the bar chart and represented the mean ± SD of three independent experiments (** *p* < 0.01, *** *p* < 0.001, ns = no significance). (**D**) Upper panels: The morphological alteration of HSC-T6 cells treated with various inhibitors including U0126, Y27632, and LY294002. The scale bar is 100 μm. Lower panels: The changes of PPARγ under exposure of different inhibitors were evaluated by Western blot analysis. The intensity of the signals was quantitated by normalizing with respect to β-actin used as internal controls. The quantified results were indicated by the bar chart and represented the mean ± SD of three independent experiments (** *p* < 0.01, ns = no significance).

To extend the details in the activation of relevant pathways and subsequent HSCs’ activation, inhibitors of ERK, PI3K/AKT, and Rho were applied. The inactivation of the ERK signal strongly suppressed the expression of p-p44/42^Thr202/Tyr204^ MAP kinase without affecting the PI3K/AKT and Rho cascades. On the other hand, the specific Rho inhibitor not only inhibited the level of correlated components such as p-Rac1/cdc42^Ser71^ but also significantly downregulated the expression of p-AKT^Ser473^, indicating that Rho/ROCK signaling plays an important role in AKT activation. Current studies have shown that PI3K/AKT contributed to cell growth and migration. AKT inhibitor treatment resulted in the blockage of p-AKT^Ser473^ and simultaneously decreased p-p44/42^Thr202/Tyr204^ MAP kinase (Figure 5A). The level of the phosphorylated signal was quantitated and normalized with respect to the intensities of the corresponding total protein (Figure 5B).

### 3.6. Interplays among ERK, AKT, and Rho Signaling Pathways and Different Vimentin Phosphorylated Sites

The phosphorylation of various vimentin sites is known to regulate their organization and function while the phosphorylation events are closely connected to the cellular processes, including proliferation and differentiation. To comprehensively explore the interaction among specific signaling pathways and phosphorylated sites of vimentin, the expression of p-VIM^Ser39^, p-VIM^Ser56^ and p-VIM^Ser83^ was detected under the administration of inhibitors of ERK, AKT, and Rho. Blocking ERK1/2 signaling using U0126 impaired the phosphorylation of p-VIM^Ser56^ while the Rho inhibitor, Y27632, particularly inhibited phosphorylation on VIM^Ser39^. At the same time, p-VIM^Ser83^ was significantly suppressed by the AKT inhibitor (Figure 5C). We also observed that the diminishment of different signaling pathways by inhibitors would notably change the morphology and differentiated status of HSCs. U0126 attenuated the cell proliferation and the Rho inhibitor altered the phenotype of the stress fiber in the HSCs. Interestingly, AKT suppression resulted in the overproduction of PPARγ and the morphology has transformed to“lipid-storing”cells in the presence of the AKT inhibitor (Figure 5D). These data reveal an integrative mechanism by which the cross talk between signaling cascades and the vimentin network may regulate the activation of the HSCs.

## 4. Discussion

HSCs exhibit activation that transforms the quiescent cells into proliferative, fibrogenic, and contractile myofibroblasts during liver fibrogenesis [30,31]. However, the intracellular signaling pathways governing activation-mediated changes in the HSCs’ morphology and motility are still unclear. Several studies have shown that the cytoskeletal system, including actin, microtubules, and IF protein such as vimentin, which is considered a hallmark of EMT, closely correlates with cell shape and motile behavior [32,33]. In the current study, vimentin was recognized as the key target for coordinating cell growth and migration during the activation of the HSCs.

The hepatic carcinogenic and mutagenic properties of DMN resulted in some liver pathological characteristics such as liver fibrogenesis and lymphocyte infiltration in a rat model [20,34]. Meanwhile, the gene and protein levels of the fibrotic markers, including α-SMA, vimentin, and collagen, were simultaneously increased along with the progression of hepatic fibrogenesis. Interestingly, vimentin was chiefly observed in the activated HSCs caused by DMN application, suggesting the pivotal role of vimentin in the changes in cell shape, adhesion, and migration that occur during HSC activation. On the other hand, in areas of non-fibrotic liver tissue, vimentin immunoreactivity was almost negative. Again, our results also showed that vimentin was significantly upregulated in the plasma samples obtained from patients with liver fibrosis or cirrhosis compared to those in the control group (*p* < 0.001). Current studies have revealed that vimentin is not only a feasible marker of epithelial to mesenchymal transition (EMT), but it also performs as a fundamental cytoskeletal protein and integrates external stimuli according to its dynamic property. In this regard, overproduction of vimentin may function as a structural scaffold and signaling system for HSC stimulation.

To further determine the functional roles of vimentin in modulating HSC activation, vimentin depletion with siRNAs was applied. Our results revealed that vimentin knockdown resulted in significant inhibition of PCNA and an increase of PPARγ, suggesting that vimentin would stimulate the proliferation and transdifferentiation of HSCs. In addition, the invasive and migratory abilities of HSCs were suppressed after siVIM application, indicating that HSC movement is clearly dependent upon the vimentin network. Moreover, the ablation of vimentin also impaired the HSCs’ contractile capacities. This correlated with our findings that vimentin-negative cells showed reduced stress fiber and aberrant filopodia in migrating HSCs. In addition, we also detected that the loss of the functional vimentin network caused the breakdown of the focal adhesion that couples the ECM to the actin cytoskeleton during cell migration. The Western blot experiments indicated that the ablation of vimentin caused marked reductions in cytoskeletal proteins including filamin, α-actinin, plectin, talin, and vinculin. Vimentin and actin are associated with integrins, through which the cytoskeletal proteins such as vinculin and plectin are recruited to induce Rho activity and act as a scaffold for proteins associated with the AMP-activated protein kinase signaling pathways [14,35,36,37,38]. Accordingly, our results also showed that vimentin deletion led to a great reduction in the activity of phos-p44/42^Thr202/Tyr204^ kinase and the Rho signaling pathway. These observations emphasize the critical role of vimentin in HSC proliferation and motility.

The activation of quiescent HSC is a complex process comprising cell proliferation, transformation, movement, and ECM production, with each of these steps being triggered by various signaling pathways [10,39]. Additionally, many reports have shown that the vimentin assembly and functions are regulated by phosphorylation, and multiple phosphorylation sites have been identified on vimentin [16,40,41]. In this regard, we utilized inhibitors against specific signaling cascades to delineate the interaction as well as the regulation between vimentin and the intracellular signaling pathways. Using an inhibitor on ERK, we demonstrated that the ERK signaling pathway might be significantly involved in controlling the proliferative response of HSCs without affecting the Rho and AKT cascades via modulating p-VIM^Ser56^, whereas AKT blocking significantly suppressed both the ERK and AKT pathways, which subsequently induced the expression of PPARγ. Similarly, we observed that the treatment of the AKT inhibitor induced the activated HSC to acquire certain properties as a “fat-storing” cell [42,43], indicating that aberrant AKT signaling could regulate the differentiated status of HSCs through inhibiting cell proliferation and reverse HSC activation at the same time. Western blotting results also showed that inhibited AKT mediated by an inhibitor could downregulate the level of p-VIM^Ser83^.

Vimentin’s enhancement of cell migration included the formation of a leading edge, lamellipodia extension, adhesion, and retraction of the trailing edge [44,45]. Cdc42 and Rac1 belonging to the Rho network are considered to be connected to the mobile ability of HSCs [46,47,48]. Furthermore, little is known about the relationship between the Rho signal and the specific sites of vimentin phosphorylation. Hence, a Rho inhibitor was applied. The results demonstrated that both the Rho and PI3K/AKT cascades were significantly arrested through the changing phosphorylation of VIM^Ser39^, which has been proven to induce vimentin filament reassembly and retraction in cells for serine phosphorylation on vimentin. It can be assumed that the upstream inhibition of Rho caused a serine phosphorylation-dependent collapse of the vimentin network, thereby disrupting the HSCs’ migration and liver fibrogenesis. This conclusion was consistent with the observation that the Rho inhibitor resulted in the generation of non-phosphorylated vimentin and displayed extensive filamentous distribution.

## 5. Conclusions

**Figure 6:**
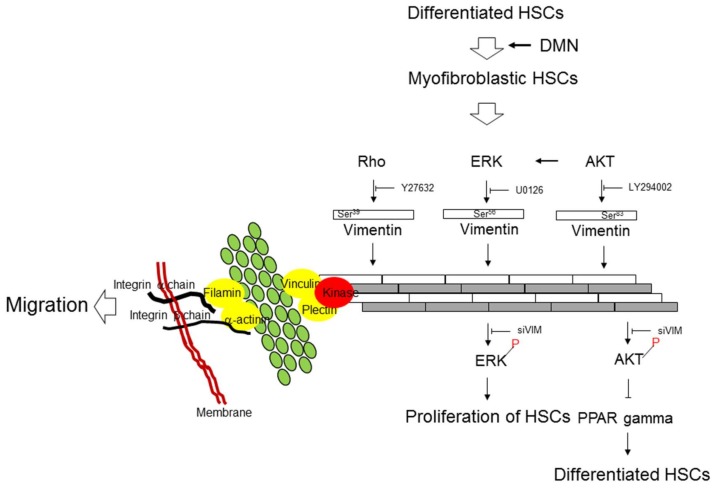
Schematic diagram indicates that the interaction between vimentin and specific signaling pathways are critically modulating the proliferation, differentiation, and migration of HSCs during liver fibrosis through the particular phosphorylated sites of vimentin.

In the current research, we have provided evidence that vimentin is the key factor in HSC activation due to its regulatory function in cell proliferation and motility. The ERK pathway is connected to HSC growth through the phosphorylation of VIM^Ser56^. Rho signaling is closely associated with migration and vimentin assembly via the phosphorylation of VIM^Ser39^, and the AKT signaling cascade is linked to HSC transformation by phosphorylation on VIM^Ser83^ (Figure 6). Vimentin also provides a major architecture for maintaining the stability of cytoskeletal proteins, which is essential for modulating the biological functions of HSCs. Our findings provide a better understanding of the intracellular signaling pathways and possible mechanisms behind the activation of HSCs. Interventions targeted at blocking the effects of these critical molecules may offer a therapeutic strategy for treating hepatic fibrosis.

## Supplementary Materials

The following are available online at https://www.mdpi.com/2073-4409/8/10/1184/s1, Figures S1 and S2.

## Abbreviations

α-SMA: α-smooth muscle actin; BSA: Bovine serum albumin; Col I: Collagen type I; Col III Collagen type III; DAPI: 4,6-diamidino-2-phenylindole; DMN: Dimethylnitrosamine; ECM: Extracellular matrix; EMT: Epithelial to Mesenchymal Transition; ERK: Extracellular-signal regulated kinase; FBS: Fetal bovine serum; GAPDH: Glyceraldehyde-3-phosphate dehydrogenase; HSCs: Hepatic stellate cells; IF: Intermediate filament; H/E: Hematoxylin–eosin; MT: Masson’s trichrome; PAKs: P21-activated kinases; PBS: Phosphate-Buffered Saline; PCNA: Proliferating cell nuclear antigen; PDGF: Platelet-derived growth factor; PPARγ: Peroxisome proliferator activated receptor-γ; RT-PCR: Reverse transcription- polymerase chain reaction; siVIM: Knockdown of vimentin with RNA silencing; TBST: Tris-buffered saline-Tween 20; TGF-β1: Transforming growth factor-β1.
