# Supplementary material for: Characterization of the Roles of Vimentin in Regulating the Proliferation and Migration of HSCs during Hepatic Fibrogenesis
Source: Cells. 2019 Oct 1;8(10):1184. doi: 10.3390/cells8101184 (PMC6830351; doi:10.3390/cells8101184)
Supplement: Supplementary file 1 [file cells-08-01184-s001.pdf]

Supplement Figure 1

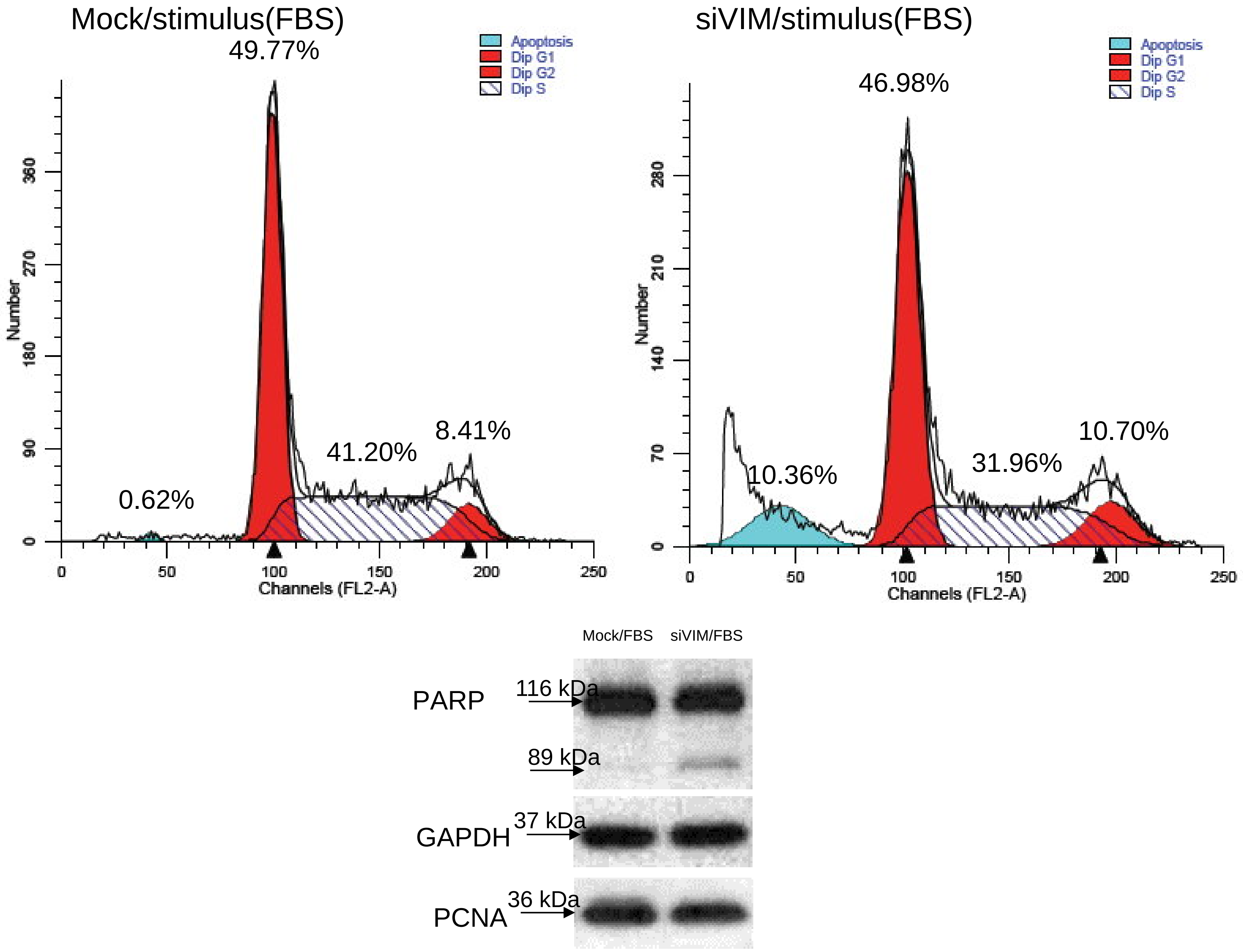

Supplement Figure 2

- Rat Liver

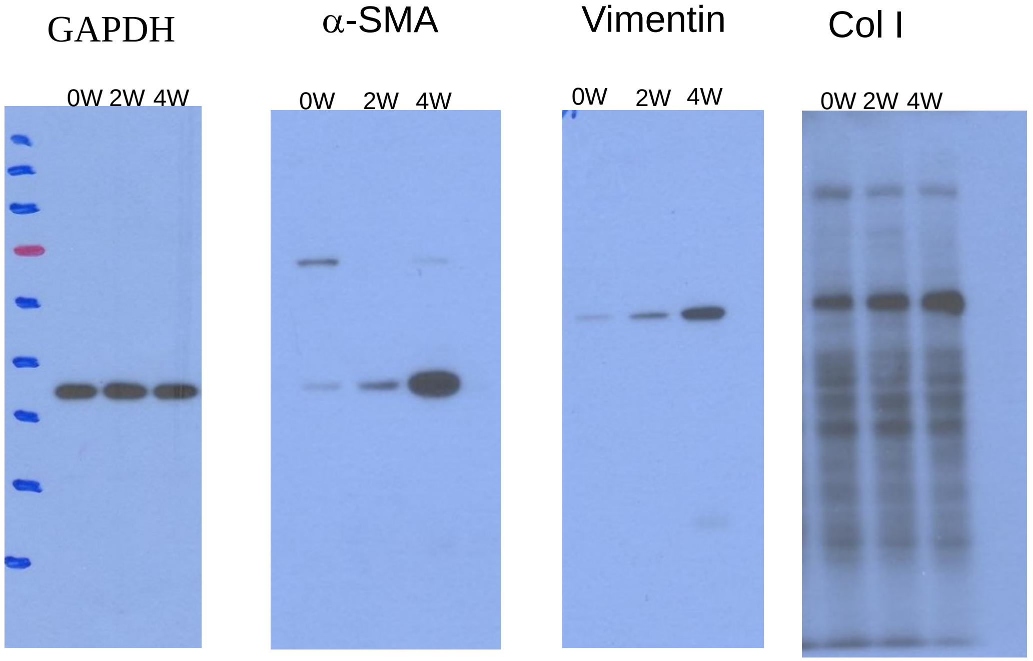

- HSC-T6

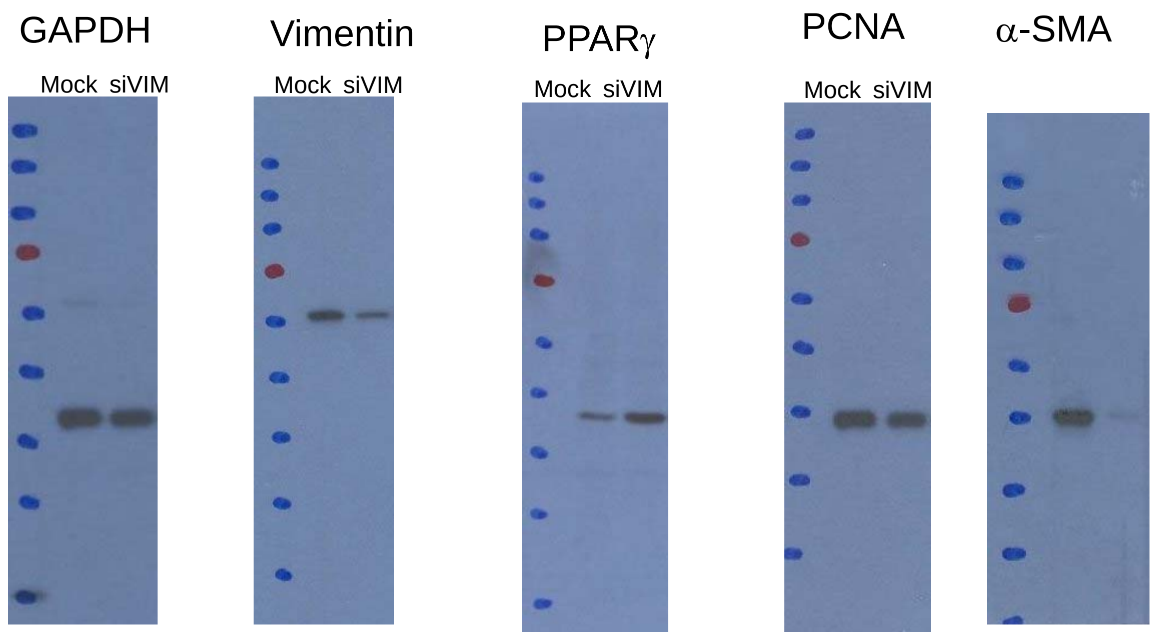

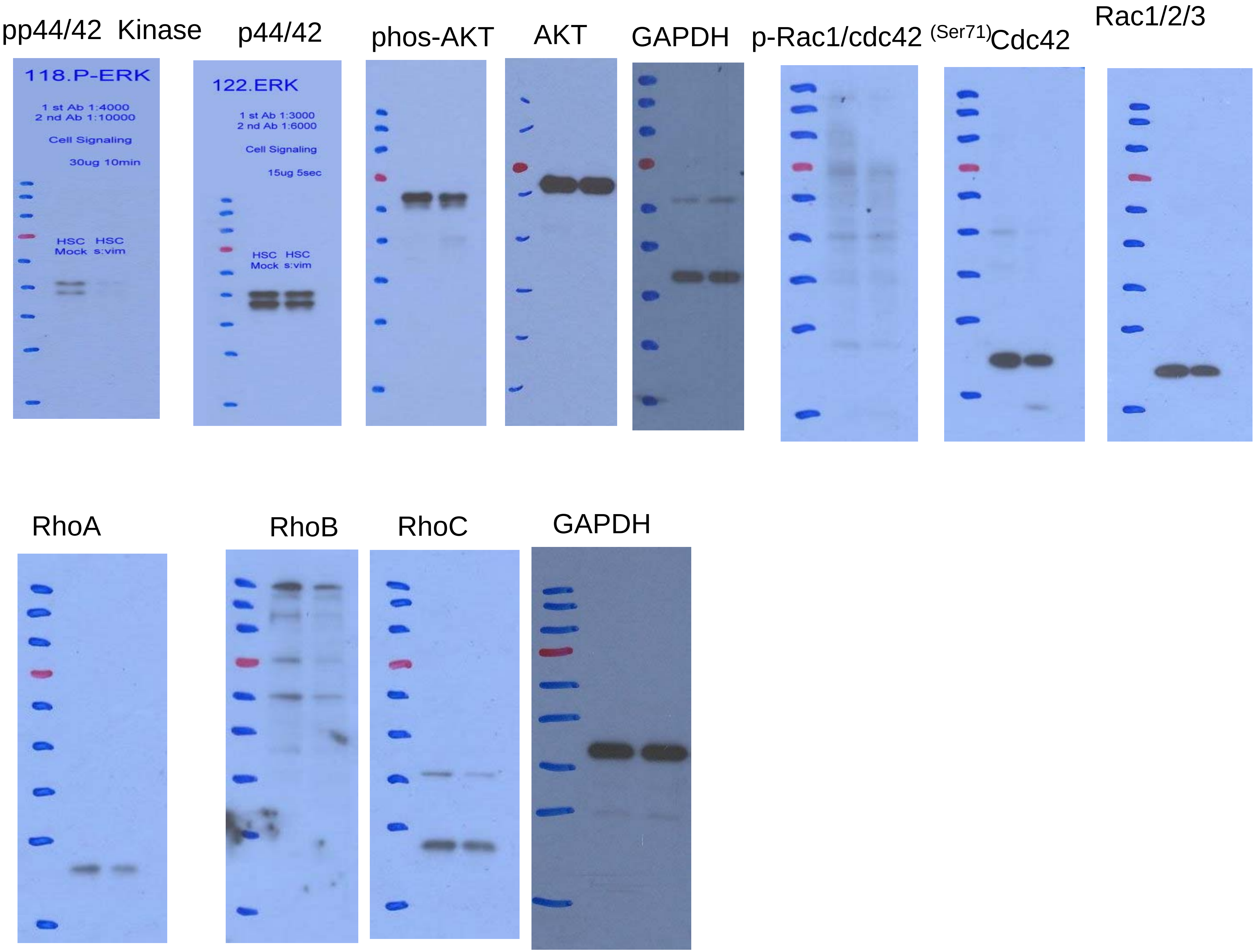
pp44/42 Kinase
118.P-ERK
1 st Ab 1:4000
2 nd Ab 1:10000
Cell Signaling
30ug 10min
HSC Mock
HSC s:vim
p44/42
122.ERK
1 st Ab 1:3000
2 nd Ab 1:6000
Cell Signaling
15ug 5sec
HSC Mock
HSC s:vim
phos-AKT
AKT
GAPDH
p-Rac1/cdc42
(Ser71)Cdc42
Rac1/2/3
RhoA
RhoB
RhoC
GAPDH

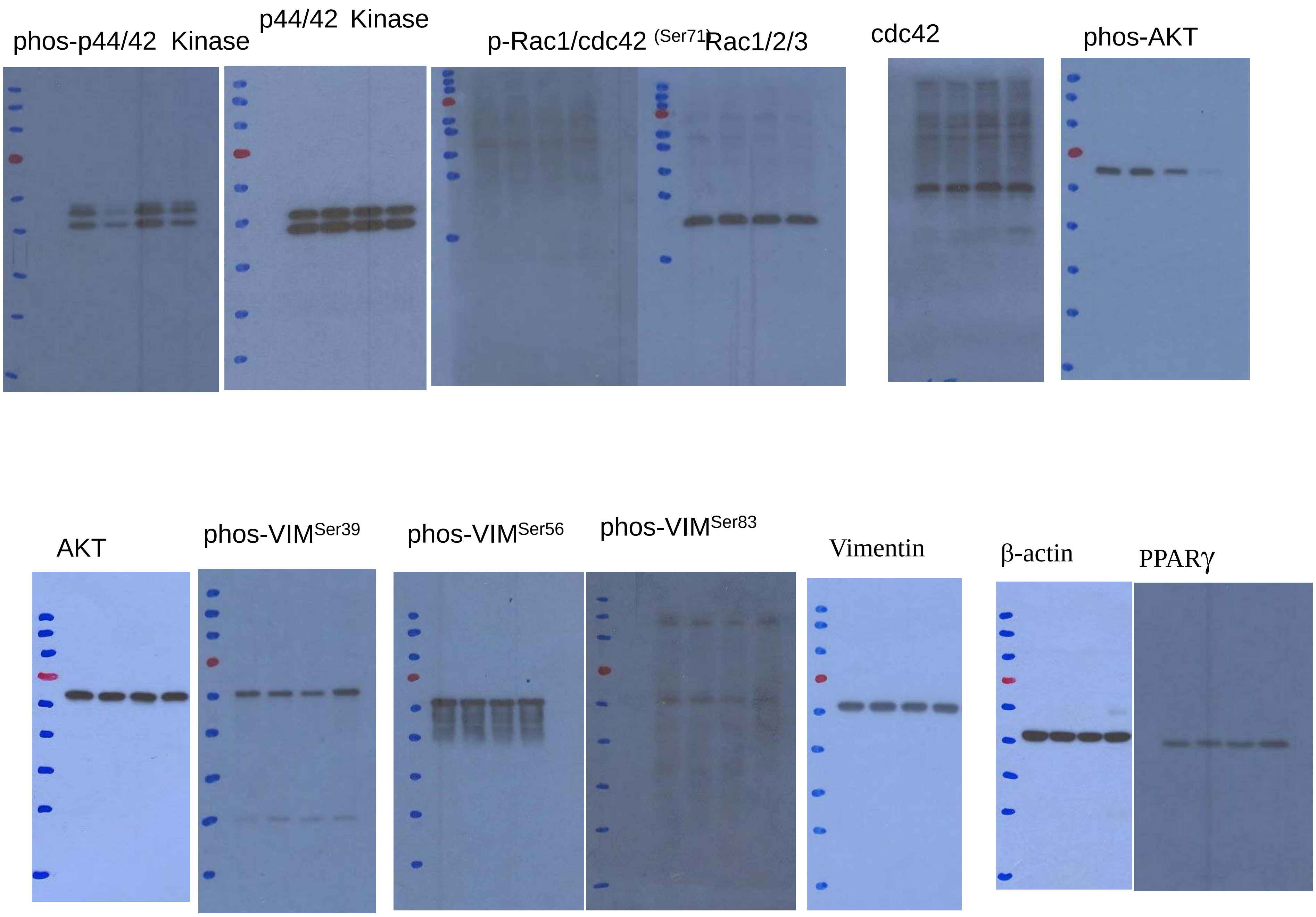
phos-p44/42 Kinase
p44/42 Kinase
p-Rac1/cdc42 (Ser71)
Rac1/2/3
cdc42
phos-AKT
AKT
phos-VIMSer39
phos-VIMSer56
phos-VIMSer83
Vimentin
β-actin
PPARγ
